# A Phospholipase C-Like Protein From *Ricinus communis* Increases Hydroxy Fatty Acids Accumulation in Transgenic Seeds of *Camelina sativa*

**DOI:** 10.3389/fpls.2018.01576

**Published:** 2018-11-01

**Authors:** Niranjan Aryal, Chaofu Lu

**Affiliations:** Department of Plant Sciences and Plant Pathology, Montana State University, Bozeman, MT, United States

**Keywords:** *Camelina sativa*, castor, hydroxy fatty acids, phospholipase C-like, triacylglycerol

## Abstract

There have been strong interests in producing unusual fatty acids in oilseed crops to provide renewable industrial feedstock. Results are so far largely disappointing since much lower amounts of such fatty acids accumulate in genetically engineered seeds than in their original natural sources. It has been suggested that the flux of unusual fatty acids through phosphatidylcholine (PC) represents a major bottleneck for high accumulation of such fatty acids in triacylglycerol (TAG). We show here that a phospholipase C-like protein (RcPLCL1) from castor bean, which accumulates nearly 90% of the hydroxylated ricinoleic acid in its seed TAG, increases the amount of hydroxy fatty acids (HFAs) when co-expresses with the fatty acid hydroxylase (RcFAH12) in transgenic seed of *Camelina sativa*. RcPLCL1 shows hydrolyzing activities on both PC and phosphatidylinositol substrates in our *in vitro* assay conditions. The PC-PLC activity of the RcPLCL1 may have increased the efficiency of HFA-PC to diacylglycerol conversion, which explains our observation of increased HFA contents in TAG concomitant with decreased HFA in the membrane lipid PC during seed development. Consequently, this may also alleviate the potential detrimental effect of HFA on germination of the engineered camelina seeds. Our results provide new knowledge that will help design effective strategies to engineer high levels of HFAs in transgenic oilseeds.

## Introduction

Triacylglycerols (TAGs) accumulated in seeds represent an important source for human consumption and industrial uses (Lu et al., 2011). Hydroxy fatty acids (HFAs) such as ricinoleic acid (12-hydroxyoctadec-cis-9-enoic acid; 18:1OH) and its derivatives are unusual fatty acids found in the seeds of castor (*Ricinus communis*) and a few other plant species such as lesquerella (*Physaria fendleri*), *Agonandra brasiliensis* and *Poliothyrsis sinensis* ^1^ (Atsmon, 1989; Snapp and Lu, 2013; Horn et al., 2016; Li et al., 2017). HFAs are a very valuable renewable resource for the chemical industry (Mutlu and Meier, 2010), however, native plants like castor and lesquerella have several drawbacks which limit their commercial production (Dierig et al., 2011; Severino et al., 2012). The hazardous chemical compounds such as ricin toxin, allergens, and ricinine are a major concern for wide castor plantation (Severino et al., 2012). Lesquerella is still being domesticated as an alternative crop for arid regions in the United States, several agronomic challenges such as weed control still remain to be surmounted for the crop to be commercialized (Dierig et al., 2011). Production of such unusual fatty acids in transgenic oilseed crops may provide a vital alternative.

Despite decades of research, unusual fatty acids only accumulate to limited amounts in transgenic seeds that are far below the levels found in their native species (Snapp and Lu, 2013; Horn et al., 2016). Transgenic Arabidopsis and camelina (*Camelina sativa*) expressing the castor fatty acid hydroxylase (RcFAH12) only produced 10–17% of HFAs in seeds, compared to nearly 90% in the castor oil (Lu and Kang, 2008; Lu et al., 2006; Snapp et al., 2014). By contrast, engineering medium-chain fatty acids (MCFAs) represents one of the few relatively successful efforts as lauric acid (12:0) can accumulate to nearly 60% of the TAGs in transgenic rapeseed by introducing an acyl-acyl carrier protein thioesterase, which terminates the fatty acyl chain elongation in the plastid (Voelker et al., 1996). The unusual acetyl-TAGs may also be accumulated to 70% of seed oils in transgenic camelina and soybean expressing an *Euonymus alatus* diacylglycerol acetyltransferase (EaDAcT), which adds an acetate instead of a long-chain fatty acid at the *sn*-3 position on diacylglycerols (Liu et al., 2015). Unlike MCFA- and acetyl-TAGs, HFAs are produced by modifying fatty acids (e.g., oleic acid; 18:1) esterified on the membrane lipid phosphatidylcholine (PC) before being incorporated into TAG. In castor, this is catalyzed by an endoplasmic reticulum-located fatty acid hydroxylase RcFAH12, a homolog of the fatty acid desaturase2 (FAD2) (van de Loo et al., 1995). Similarly, other divergent forms of FAD2 catalyze the introduction of triple or acetylenic bonds, epoxy groups, conjugated and *trans* double bonds on C18-fatty acids on PC (Jaworski and Cahoon, 2003). Therefore, an inefficient flux of HFA and other unusual fatty acids from PC and subsequent incorporation onto TAG presents a significant bottleneck for the accumulation of high amounts of such fatty acids in transgenic seed oils (Bates and Browse, 2011). Consequently, this inefficiency may also inhibit fatty acid synthesis that results in low oil content in transgenic seeds (Bates and Browse, 2011; Bates et al., 2014), and affects seed vigor with low germination ability (Dauk et al., 2007). Modified fatty acids on PC may enter the acyl-CoA pool for TAG assembly by the so-called acyl editing enzymes including lysophosphatidylcholine acyltransferases (LPCAT), acyl-CoA:glycerophosphocholine acyltransferases (GPCAT), and phospholipase A2 (PLA2) (Bates et al., 2012; Bayon et al., 2015; Lager et al., 2015). PC may also be converted to diacylglycerol (DAG), the immediate precursor of TAG, by the headgroup exchange using the phosphatidylcholine:diacylglycerol cholinephosphotransferase (PDCT) (Lu et al., 2009). In addition, phospholipid:diacylglycerol acyltransferases (PDAT) may directly transfer acyl groups from PC to DAG and form TAG (Dahlqvist et al., 2000). PDCT, PDAT1A and PLA2α originated from castor had been shown to be involved in removing HFA from PC (van Erp et al., 2011; Hu et al., 2012; Bayon et al., 2015). By co-expressing with *RcFAH12*, these genes significantly affected HFA accumulation in transgenic seed, and at least partly restored the oil content and seed germination potential (Lu et al., 2006; Burgal et al., 2008; van Erp et al., 2011; Hu et al., 2012; Snapp et al., 2014). Engineering enzymes involved in PC metabolism thus provides a potential strategy for high accumulation of HFA and other unusual fatty acids in transgenic oilseeds (Snapp and Lu, 2013).

Phosphatidylcholine:diacylglycerol cholinephosphotransferase and LPCAT direct nearly two-thirds of 18:1 toward PC for desaturation and subsequent incorporation of the polyunsaturated 18:2 + 18:3 into TAG (Lu et al., 2009; Bates et al., 2012). The rest of the 18:1 modification through PC are suggested to be carried out by enzymes in the Kennedy pathway (Wang et al., 2012). Phospholipase C (PLC) is a major membrane phospholipid hydrolyzing enzyme. In plants, the PLC class has been divided into three groups according to their substrates: a well-studied phosphatidylinositol-specific PLCs (PI-PLCs), non-specific PLCs (NPC) that acts on common phospholipids such as PC and phosphatidylethanolamine (PE), and the glycosylphosphatidylinositol (GPI)-PLC that hydrolyzes GPI-anchored proteins (Wang, 2001). Their reactions yield diacylglycerols (DAG) and phosphorylated head-groups (Wang, 2001). The roles of PLC, especially PI-PLC, in lipid signaling have been extensively studied (Hong et al., 2016), however, very little is known in storage lipid metabolism. In this research, we discovered that a putative PLC-like protein that was previously shown to be highly expressed in castor endosperm increased HFA accumulation in transgenic camelina seed when co-expressing with the RcFAH12.

## Materials and Methods

### Plant Material and Green House Growth Condition

The *C. sativa* line RcFAH (#7-1) was produced previously by transforming a castor fatty acid hydroxylase (RcFAH12) under a seed-specific phaseolin promoter and the DsRed fluorescent marker (Lu and Kang, 2008). An untransformed camelina cultivar Suneson with the same genetic background as #7-1 was used as the wild type plant line. Four homozygous RcPLCL1 transgenic lines showing strong effect on HFA accumulation were used for characterization. Plants were grown in a 6″ or 8″ pot containing a 1:1 mix of MSU soil (equal parts by volume of loam soil:washed concrete sand:Canadian sphagnum peat moss with AquaGro 2000 G wetting agent blended in at 1 lb/cubic yard of soil. Aerated steam pasteurized at 70°C for 1 h) and Sunshine Mix #1 (Bellevue, WA, United States). Greenhouse conditions were 22°/18°C + /-1°C for day/night temperatures, a relative humidity of 30%, and a 16-h photoperiod of natural lighting supplemented when necessary by season.

### Plasmid Construction and Plant Transformation

A fragment of ∼1.2 Kb of the putative *RcPLCL1* gene was amplified from the castor cDNA library constructed previously (Lu et al., 2006) using primers ATGTTTGCGTGCTTCGCGGACTACTGTA and TAATAAAGATGCCATTAGTGAAA, and inserted into a binary vector, PinGlyBar1 (Nguyen et al., 2013), at the restriction sites *Eco*RI and *Xho*I between the soybean Glycinin promoter and Glycinin terminator. The vector contained Kanamycin resistance for the *E. coli* selection and *bar1* for transgenic plant selection. The plasmid was transformed into the Agrobacterium strain GV3101, and transgenic plants were obtained by vacuum infiltration method as described (Lu and Kang, 2008). Seed from T0 plants was harvested, and then planted directly in flats to screen transgenics by spraying with glufosinate herbicide at a concentration of 23.4 ml/L (5.78% glufosinate ammonium, Power Force Grass and Weed Killer, Bayer, Birmingham, AL, United States). Surviving plants were also tested for the presence of the *RcPLCL1* gene by PCR using the above gene specific primers. The individual plants showing the highest HFA amount were advanced to the T_4_ generation to obtain homozygous lines.

### Lipid Extraction and Fatty Acid Analysis

Fatty acid methyl esters (FAMEs) were created from the total lipids of the mature seeds using: (1) TMSH method: Trimethylsulfonium hydroxide (TMSH) preparation was used for the 96-well plate screenings as described previously (Lu et al., 2006). (2) Acid derivation method: FAMEs were derived from seeds in 1 ml of 2.5% sulfuric acid in methanol at 80°C for 90 min (Browse et al., 1986). FAMEs were injected into a Shimadzu 2010 GC fitted with a narrow bore column (HP-Innowax; 30 m × 0.25 mm i.d. × 0.25 μm; Agilent Technologies). The oven temperature was programmed at 190°C initially followed by an increase of 20°C/min to 250°C and maintained for 9 min.

For stereochemical analysis, total lipids were extracted from a total of 15 seeds using a modified Blight and Dyer method (Hu et al., 2012). HFA-containing TAG (1OH TAG, 2OH TAG) and normal TAG were then separated by thin layer chromatography (TLC) on silica gel plates (silica gel 60, 20 cm × 20 cm, EMD Chemicals, Darmstadt, Germany) using the solvent system of 70:30:1 v/v Hexane:anhydrous ethyl ether:formic acid (Snapp et al., 2014). The separated bands were scrapped off the TLC plates and the above acid derivative method was used to analyze FAMEs by GC. Phospholipids (mainly PC) that remained at the sample loading spots on the TLC plates were also analyzed by GC.

### Enzyme Activity Assay

Genes for *RcPLCL1* and *AtPLCL1* (At5g67130) were amplified from cDNAs of castor endosperm and Arabidopsis, respectively. The primers for *AtPLCL1* were ATGTCGGCGTGCATCAATGGC and TAGTAGAAATATCAGCAACGG. The PCR products were cloned into the pGEMT-Easy vector (Promega, Madison, WI, United States) and sequenced for the confirmation of complete and correct sequences. Then the *RcPLCL1* and *AtPLCL1* were cloned into the pYES2 expression vector at the *Eco*RI and *Xho*I sites and transformed into InvSc1 expression yeast cells by lithium acetate method (Kang et al., 2011). The yeast culture of 300 ml was grown by shaking at 30°C for 24 h in SD-Ura medium containing 2% galactose. Microsomal preparation and enzyme assay were performed as described previously (Lu et al., 2009). Cells were harvested by centrifugation at 2000 × *g* for 10 min. The pellet was washed with sterile water. Washed cells were then resuspended in ice-cold glucose-Tris-EDTA (GTE) buffer [20% glycerol, 50 mM Tris–HC1 (pH 7.4), 1 mM EDTA]. Freeze and thaw method was used for releasing the membrane proteins. Solution was frozen using liquid nitrogen for 3 min and then thawed rapidly at 42°C. The process was repeated for three times. The solution was then centrifuged at 2000 × *g* for 10 min and then the supernatant was collected for further enzyme assay. The microsomes thus obtained were quantified by running a protein gel and by measuring with the NanoDrop at 280 nm wavelength. About 5–10μg of protein was used for the enzyme assay. The substrates 1,2-dipalmitoyl-*sn*-glycero-3-phosphocholine (di16:0 PC) and 1,2-dipalmitoyl-*sn*-glycero-3-phosphoinositol (di16:0 PI) were used. Substrates were dried under nitrogen gas and resuspended in reaction buffer [final concentrations: 50 mM 3-(N-morpholino) propanesulfonic acid (MOPS)/NaOH (pH 7.5), 20 mM MgCl2, 0.45% Triton X-100]. Microsomes suspended in GTE buffer was added and reaction volume was made to 200μl using reaction buffer. The reaction was performed at 15°C for 1 h, and stopped by adding 1 ml of chloroform:ethanol :: 2:1 and 1.5 ml of 0.9% NaCl. The mixture was centrifuged at 2000 × *g* for 5 min and the upper layer was dried with nitrogen gas and dissolved in 100 μl of chloroform. Lipids were separated by TLC using hexane:ethyl ether:formic acid (70:30:1) as solvents. The origin (PC or PI) and the DAG bands were recovered and analyzed using gas chromatography by creating FAMEs.

### Oil Content Measurement and Germination Test

Seed oil content was determined by a bench-top NMR seed analyzer (MQC23, Oxford Instruments, Concord, MA, United States) and by GC analysis using heptadecanoic acid (17:0) as internal standard (10 mg/ml), which was added to test tubes prior to FAMEs derivatization.

For germination tests, 100 seeds from each line were placed on wet filter papers in covered petri dishes. Seeds were left to germinate for 7 days at room temperature. Germination was determined by radicle emergence.

### Phylogenetic Analysis

A neighbor-joining tree was made using MEGA6 (Tamura et al., 2013). Sequences for different PLCs were obtained from NCBI^2^ and the Arabidopsis TAIR^3^ websites. The sequences were then aligned by ClustalW using the default parameters. Unaligned long flanking sequences were chopped off for both sides and a neighbor joining tree was built.

## Results

### Sequence Analysis of a Putative Phospholipase C-Like Gene (*RcPLCL1*) That Was Highly Expressed in Castor Endosperm

**FIGURE 1:**
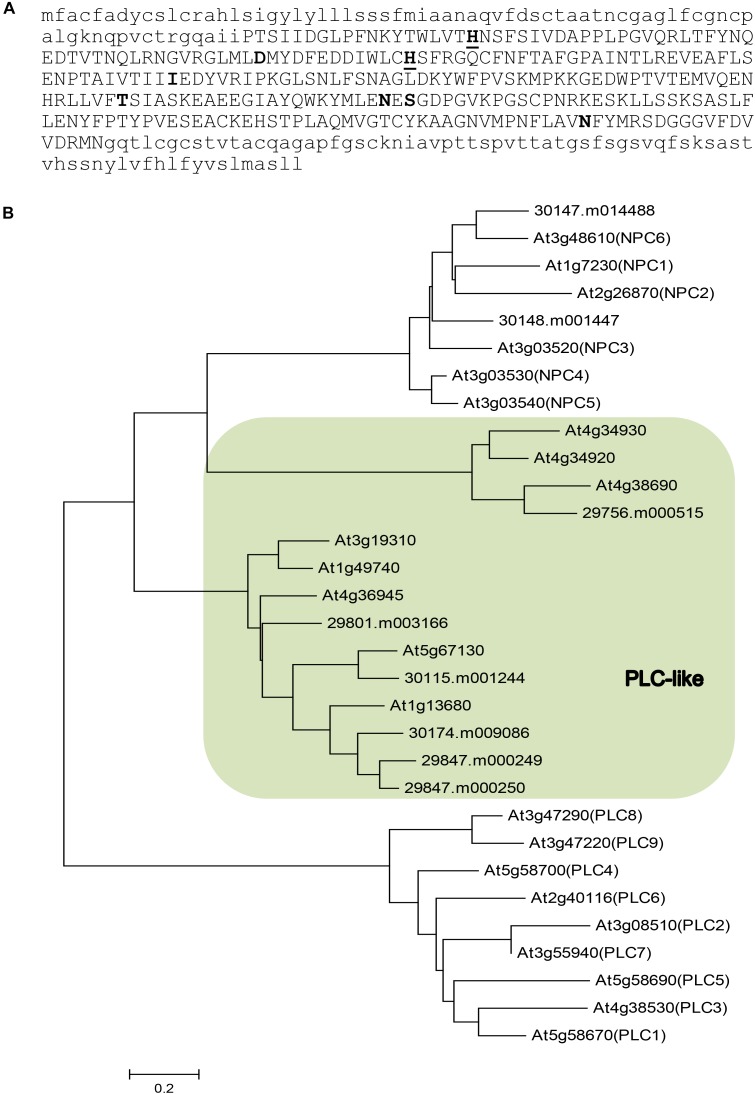
Sequence analysis of putative phospholipase Cs in castor (*Ricinus communis*) and their homologs in Arabidopsis. **(A)** Amino acid sequence of RcPLCL1 (30115.m001244). The PI-PLC X domain is shown in upper case letters. Active histidine (underlined) and putative active sites are shown in bold. **(B)** A neighbor-joining tree for putative phospholipases in castor and their homologs in Arabidopsis. Shaded area indicates genes encoding putative PLC-like proteins.

To identify phospholipase Cs that may be involved in TAG synthesis especially HFA accumulation, we examined a previous report on tissue-specific transcriptome sequencing in castor (Brown et al., 2012). In that study, eight genes encoding putative PLCs were found to show significant differential expression in castor tissues (Supplementary Table S1). Phylogenetic analysis suggests that they belong to two distinct groups (Figure 1): two genes are homologous to the Arabidopsis non-specific NPCs (30147.m014488 and 30148.m001447); six genes that we name as *RcPLCLs* encode putative PLC-like phosphodiesterases that belong to a PI-PLCc-GDPD_SF superfamily. Among the three groups of phospholipase Cs, the PI-hydrolyzing PLC is better understood (Hong et al., 2016). Plant PLCs all contain catalytic X and Y domains that form a TIM-barrel-like structure necessary for the phosphoesterase activity, followed by a Ca^2+^-dependent phospholipid binding C2 domain at the C terminus (Wang, 2001). Analyzing the protein sequences using Prosite at ExPasy^4^ suggests the castor PLC-like protein contains an X domain with two Histidine active sites (Figure 1A). However, the software did not predict Y and C2 domain of PI-PLCs in the RcPLCL protein sequences.

To investigate the roles of RcPLCL in lipid metabolism especially in HFA accumulation in TAG, we chose the *RcPLCL1* (30015.m001244) since it is the most highly expressed gene in castor endosperm, though its transcripts were also found in germinating seed, leaf, and male flowers. Other putative RcPLCLs are phylogenetically related, but they were primarily expressed in leaves and/or male flowers, and very low or no expression in developing endosperm of castor (Brown et al., 2012). A putative non-specific RcNPC6 (30147.m014488) homologous to the Arabidopsis *AtNPC6* showed similar temporal expression pattern, but higher gene expression levels were in leaf and male flowers (Supplementary Table S1), suggesting its general roles of lipid metabolism in castor besides possible functions in storage lipid accumulation.

### *RcPLCL1* Expression Increases HFA Contents in *RcFAH12*-Containing Camelina Seeds

To test the effect of RcPLCL1 on HFA accumulation in TAG, the *RcPLCL1* cDNA was cloned from a cDNA library (Lu et al., 2006) and inserted into a plant transformation vector under a strong seed-specific phaseolin promoter (Sengupta-Gopalan et al., 1985). The *pPhas:RcPLCL1* construct was then transformed into a camelina RcFAH (#7-1) line expressing the *RcFAH12* and accumulating 15% HFA (Lu and Kang, 2008; Snapp et al., 2014). The herbicide resistance conferred by the *bar* gene was chosen as the selection maker because seeds of the recipient RcFAH plants contain the DsRed marker (Lu and Kang, 2008). Meanwhile, we also made the *pPhas:AtPLCL1* (At5g67130) construct and transformed into the RcFAH line. AtPLCL1 showed the closest homolog to the RcPLCL1 (Figure 1B). We obtained a total of 13 and 14 T1 plants expressing *RcPLCL1* and *AtPLCL1*, respectively. The effects of RcPLCL1 and AtPLCL1 on HFA accumulation were estimated by analyzing fatty acid composition of 20 individual seeds from each T1 plant. The sample seeds from each T1 plant were hemizygous for the transgenes or control segregants containing the *RcFAH12* only. Results indicated that all RcPLCL1 T1 produced some seeds with significantly increased HFA contents compared to RcFAH seeds (Supplementary Figure S1). Total HFAs, including ricinoleic acid (18:1OH), densipolic acid (18:2OH), lesquerolic acid (20:1OH), and auricolic acid (20:2OH), in individual high-HFA RcPLCL1 lines ranged from 19 to 24% of the total FAs in seeds, representing about 27–60% increases from the level in the RcFAH line at 15%. By contrast, the AtPLCL1 lines did not show significant changes in seed fatty acid composition compared to the RcFAH line (Supplementary Figure S2). We did not conduct more experiments on the AtPLCL1 transgenic lines.

**FIGURE 2:**
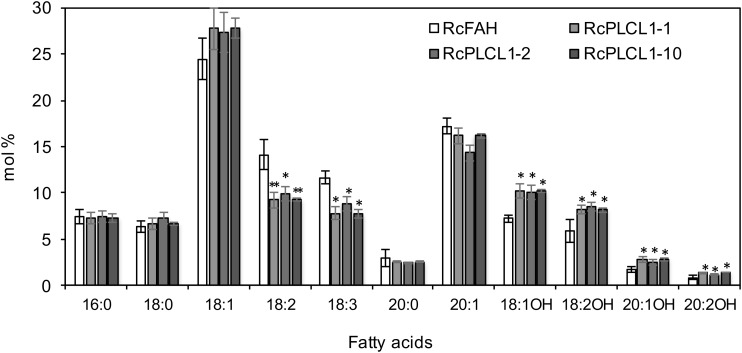
Fatty acid profiles of camelina transgenic seeds. RcFAH, Camelina expressing RcFAH12 alone; RcPLCL1, Camelina coexpressing RcFAH12 and RcPLCL1. Data are average ± SD from seeds of homozygous T3 lines showing highest HFA contents. Two-tailed Student’s *t*-test. ^∗^*P* < 0.05; ^∗∗^*P* < 0.01.

All RcPLCL1 T2 plants were grown and four homozygous T3 lines were chosen for further studies. The average seed fatty acid composition of the three best lines (#1, 2, 10) is presented in Figure 2. All species of HFAs had increased in RcPLCL1 lines compared to those in the RcFAH line, and the increased HFAs mostly came at the expenses of decreased amounts of polyunsaturated 18:2 and 18:3. We extracted total lipids from bulked RcPLCL1 and RcFAH dry seeds. HFA-containing TAGs were separated on TLC plates followed by GC analysis as described previously (Snapp et al., 2014). The 1-OH TAG and 2-OH TAG, which contain 1 or 2 hydroxy acyl-chains on the TAG molecules, showed increases from 28 and 51.3% in RcFAH to 29.7 and 54.5% in RcPLCL1, respectively (Supplementary Figure S3). These results indicated that the putative RcPLCL1 was involved in HFA metabolism and its accumulation in TAG.

### RcPLCL1 Hydrolyzes PC and PI *in vitro*

**FIGURE 3:**
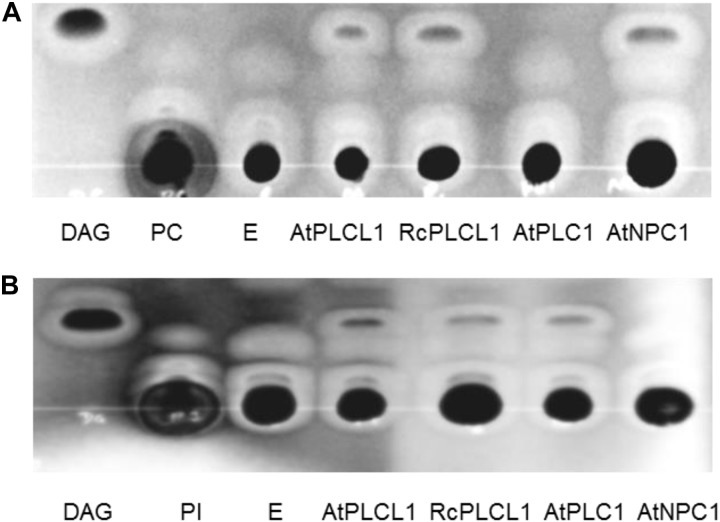
Phospholipase activity assay for RcPLCL1 and AtPLCL1. Lipids were separated on TLC plates after being extracted from reactions with substrates **(A)** 1,2-dipalmitoyl-*sn*-glycero-3-phosphocholine (di16:0 PC) or **(B)** 1-palmitoyl-2-oleoyl-*sn*-glycero-3-phosphoinositol (16:0-18:1 PI) with yeast microsomes containing genes cloned in the pYES2 vector. E, pYES2 empty vector.

Analysis in SCOP hierarchy Superfamily HMM server (Wilson et al., 2007) suggests that the RcPLCL1 may have hydrolytic functions such as phosphoric di/ester hydrolase and phospholipase activities. To determine its phospholipase activity, we tried to use the RcPLCL1 recombinant protein in *E. coli*, however, this attempt was not successful since the *6XHis-RcPLCL1* expression was very low. We then cloned *RcPLCL1* and its Arabidopsis homolog *AtPLCL1* (At5g67130) into a pYES2 vector and expressed in yeast (*Saccharomyces cerevisiae*) cells. The previously characterized *AtPLC1* (At5g58670) and *AtNPC1* (At1g07230), which specifically hydrolyzed phosphatidylinositol (PI) and PC, respectively (Hirayama et al., 1995; Krčková et al., 2015), were also expressed in yeast cells as controls. Microsomal preparations from yeast cells were incubated with 1,2-dipalmitoyl-*sn*-glycero-3-phosphocholine (di16:0 PC) and 1,2-dipalmitoyl-*sn*-glycero-3-phosphoinositol (di16:0 PI) substrates. Lipids were then extracted from reaction mixes and separated on TLC plates. As shown in Figure 3, incubation of yeast microsomes isolated from cells transformed with an empty pYES2 vector did not yield detectable products from PI or PC substrates. This result indicated that endogenous phospholipase activities of the InVSc1 cells could be neglected in our experiments. When incubated the PI or PC substrates with RcPLCL1 and AtPLCL1 containing microsomal proteins, bands migrated at the same rate as DAG were detected in both reactions. The bands were scraped and analyzed with gas chromatography for fatty acid composition, which were identical to the PI or PC substrates. These results indicated that the bands were DAG produced from PI or PC. In control experiments with AtPLC1 and AtNPC1, DAG bad was detected only when PI or PC was supplied as substrates, respectively. These results suggest that the RcPLCL1 and AtPLCL1 possess phospholipid hydrolyzing activities acting on both PI and PC substrates.

### HFA Accumulation During Camelina Seed Development

**FIGURE 4:**
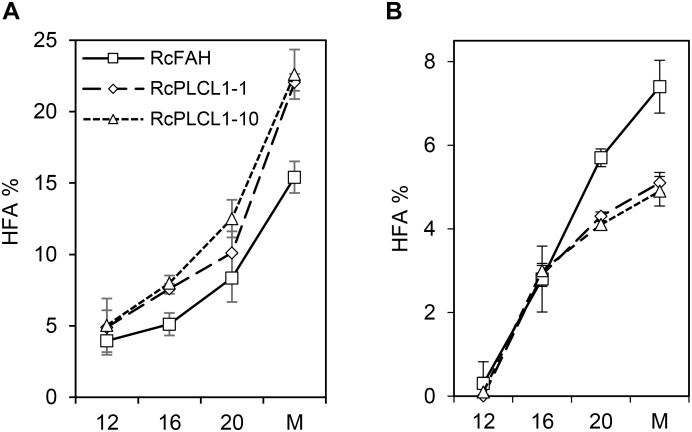
Hydroxy fatty acid (HFA) accumulation in camelina developing seeds. **(A)** Percentage of HFA in total seed oils. **(B)** Percentage of HFA in polar lipid (PC) fraction. M, Mature seeds. Data represent average ± SD of three replicates.

The increased HFA accumulation in the RcPLCL1 seed compared to RcFAH raised the possibility that the activity of RcPLCL1 may enhance the conversion of PC into DAG. To test this hypothesis, we examined HFA accumulation during seed development. As shown previously, camelina seeds rapidly synthesize storage lipids during 8–30 days after flowering (DAF) (Rodriguez-Rodriguez et al., 2013). In the RcFAH12-expressing camelina seeds, the most rapid TAG synthesis occurred during 10–20 DAF and the HFA content also steadily increased (Snapp et al., 2014; Horn et al., 2016). We excised seeds from 12, 16, and 20 DAF from three plants of each RcFAH and two RcPLCL1 lines (#1 and #10). Total oils were directly analyzed by GC using whole seeds, and PC fractions were analyzed after separating from neutral lipids on TLC plates as described previously (Snapp et al., 2014). As shown in Figure 4, HFA accounted for nearly 5% of total FAs in seed oils at 12 DAF and steadily increased as seeds matured in both groups of transgenic plants. At all developmental stages RcPLCL1 seeds contained significantly more amounts of HFAs compared to the RcFAH seeds collected at the same time points (Figure 4A). HFAs in the PC fraction also increased during seed development, however, their amounts started to differ after 16 DAF with significantly lesser accumulation found in the RcPLCL1 seeds than the RcFAH controls. In mature seeds, RcPLCL1 seeds contained ∼5% HFAs in the polar PC fraction, while the RcFAH seeds contained nearly 7.5% HFA in the PC (Figure 4B). These observations clearly indicated that RcPLCL1 increased the efficiency of HFAs removal from PC, where they were synthesized by the RcFAH12, by converting to DAG and resulted in more HFA accumulation in the TAG of the RcPLCL1-expressing camelina seeds.

### RcPLCL1 Improved Germination of HFA-Camelina Seeds

**FIGURE 5:**
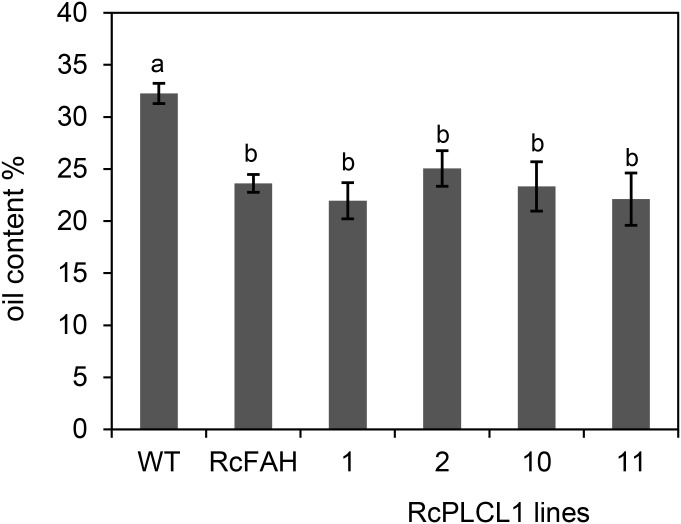
Total fatty acid content of camelina seeds. WT, non-transgenic camelina variety Suneson; RcFAH, *RcFAH12* transgenic line #7-1; 1, 2, 10, and 11 are transgenic seeds of T4 RcPLCL1 homozygous lines. Data represent average ± SD for seeds harvested from three plants of each line. One-tailed Student’s *t*-test, columns with different letters are significantly different.

To assess the effects of HFA accumulation in camelina seeds, we measured the oil content and performed germination tests of the transgenic seeds. Previous reports had shown that oil content decreased in HFA-accumulating transgenic seeds of camelina and Arabidopsis plants due primarily to the feedback inhibition during oil biosynthesis (van Erp et al., 2011; Bates et al., 2014; Snapp et al., 2014). The RcFAH camelina seeds contained nearly 30% less oil than the non-transgenic control seeds (Figure 5). The four lines of RcPLCL1-expressing seeds that we examined did not show significant differences from the RcFAH seeds. This result suggested that although it helped the flux of HFA from PC into DAG, RcPLCL1 was not able to alleviate the reduced TAG accumulation in the RcFAH transgenic seeds.

**FIGURE 6:**
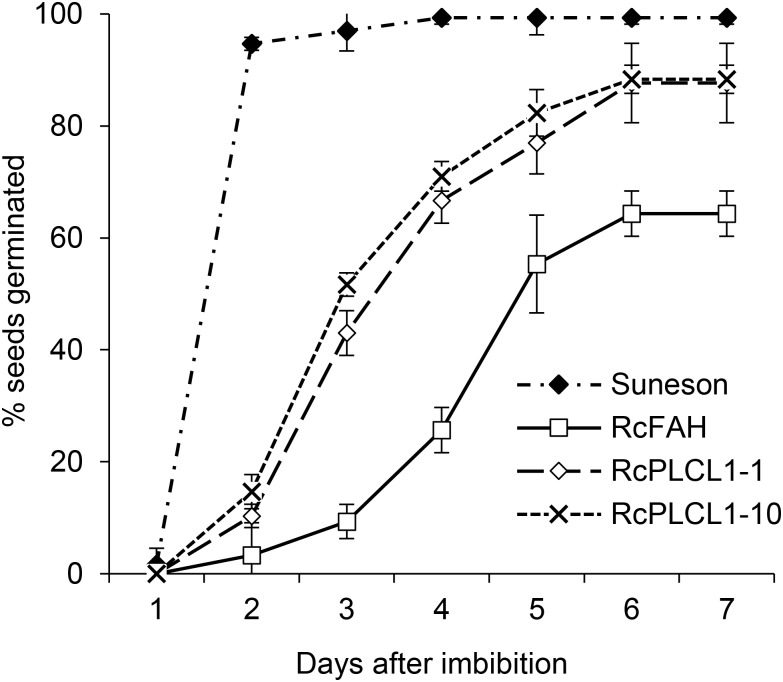
Germination rates of camelina wild type and transgenic seeds. Data are the average of three replicated experiments.

Homozygous T4 transgenic seeds of two RcPLCL1 lines (#1 and #10) were also tested for germination by plating on damp filter papers. Wild type seeds were included as controls, which mostly germinated the next day showing radicle emergence and followed by successful seedling growth. Camelina RcFAH seeds had a slower germination rate and nearly 40% failed to germinate at 7 days after imbibition. The germination rate for the RcPLCL1 seeds were improved compared to the RcFAH line. While 10–20% seeds still did not germinate during the test time-period, the RcPLCL1 seeds showed significantly increased germination rate and potential (Figure 6).

## Discussion

The castor fatty acid hydroxylase (RcFAH12) catalyzes the 18:1-PC hydroxylation on the ER (van de Loo et al., 1995). Expression of RcFAH12 in Arabidopsis or camelina resulted in limited amounts (15–17%) of HFA in transgenic seeds (Lu et al., 2006; Lu and Kang, 2008). Previous studies have indicated that the efficient flux of HFA through PC represents the major bottleneck of high levels of HFA accumulation in heterologous transgenic seeds (Bates et al., 2014), and co-expressing genes involved in PC turnover during seed development, e.g., PDCT and PDAT1A, with RcFAH12 can enhance HFA accumulation (van Erp et al., 2011; Hu et al., 2012). Phospholipases (e.g., PLC and PLD) play major roles in converting PC to DAG, thus potentially affect FA composition in TAG. In this study we tested a castor PLC like protein (RcPLCL1) and demonstrated that it had similar effects on enhancing HFA accumulation in RcFAH12-expressing camelina seed.

Multiple forms of PLCs exist in plants and they differ in their substrate specificities. PI- and non-specific PC-hydrolyzing PLCs have been extensively studied previously, but mostly focused on lipid signaling (Wang, 2001; Hong et al., 2016). The roles of PLCs in seed storage lipid biosynthesis are unclear. Previously, transcriptome analysis in castor tissues identified several putative PLCs including NPCs and a group of PLC-like proteins that are distinctly different from known PLCs (Brown et al., 2012). The castor NPC homologs would be our candidates to enhance HFA accumulation in transgenic seed since they hydrolyze PC, however, the two putative *NPC* genes are primarily expressed in leaf and male flower (Supplementary Table S1), suggesting their minor roles in storage lipid biosynthesis. We therefore tested the putative PLC-like proteins. Sequence analysis using BLAST and other software indicated that the putative PLC like proteins in castor share a catalytic X-domain of PI-PLCs and may have phosphodiesterase activities on phospholipids. Homologs of these genes are also present in other plants such as Arabidopsis. These findings suggest that PLC-like proteins may have diverse functions in plants. There are six *RcPLCL* genes in castor that are widely expressed in different tissues including leaf, male flower, developing endosperm, and germinating seed (Supplementary Table S1). For the objective of our study, we chose *RcPLCL1* because it is highly expressed in all tissues but particularly in endosperms where castor oils are stored.

Our results indicate that RcPLCL1 contributes to lipid biosynthesis during TAG accumulation in seed. The bioinformatically predicted phospholipase activity was detected in RcPLCL1 and its closest homolog in Arabidopsis (AtPLCL1). When expressed in yeast, the microsomal proteins hydrolyzed PI and PC substrates in our assay conditions. In plants, the PI-PLC activity of RcPLCL1 may produce the signaling molecules phosphoinositols that potentially regulate biochemical processes. Though we could not rule out this possible effect on HFA metabolism, it is more likely that the PC-hydrolyzing activity of RcPLCL1 was responsible in transgenic camelina that enhanced the HFA accumulation in seed TAG by converting HFA-containing PC into HFA-DAG. This was supported by our observation that HFA contents in PC declined concomitantly with their increases in TAG during seed development of camelina co-expressing RcPLCL1 and RcFAH12.

In castor, the majority of ricinoleic acid may be released from PC after 18:1 hydroxylation and enter the acyl-CoA pool to participate in TAG biosynthesis through the Kennedy pathway. To some extent, ricinoleic-PC also may turn into DAG through phosphocholine headgroup exchange (Bafor et al., 1991). In transgenic Arabidopsis and camelina, a major pathway for HFA-TAG biosynthesis is reported through the PC-DAG interconversion (Bates and Browse, 2011, 2012). Previously we showed that PDCT is required for fatty acid hydroxylation in RcFAH-transgenic Arabidopsis and coexpressing a castor RcPDCT with RcFAH increased HFA accumulation (Hu et al., 2012). Here we show that a PLC-like protein also increased total HFA content in RcFAH-expressing camelina seeds, accordingly the 1-OH-TAG and 2-OH-TAG fractions were also increased. These results suggest that, besides RcPDCT, RcPLCL1 is also involved in the conversion of PC to DAG. It is not clear whether the increase of HFA in TAG is caused by a general enhancement of PC to DAG conversion, but it is possible that the RcPLCL1 may specifically act on HFA-containing PC. This type of substrate specificity has been demonstrated in caster genes, e.g., RcPDAT1A, RcDGAT2, RcPLA2a (Burgal et al., 2008; van Erp et al., 2011; Bayon et al., 2015). In our parallel experiment, an Arabidopsis homolog AtPLCL1 with 69% amino acid identity failed to increase HFA in the RcFAH-containing seed. This result suggests a similar substrate specificity of RcPLCL1. However, we are not able to draw such a conclusion without supports with more enzyme activity assays and transgenic experiments using additional homologous *AtPLCL* genes. The PLC-like phosphoesterases isolated in this study have not been characterized previously. Their homologous genes are widely present in other plants like Arabidopsis (Figure 1). More research is needed to understand the full spectrum of enzyme activities and biological roles of this class of phospholipases in plant development and metabolism.

The biotechnological challenges of producing HFAs in transgenic oilseeds include the detrimental effects on seed physiology such as decreased seed viability and oil accumulation (Snapp and Lu, 2013). RcPLCL1 effectively removed HFA from the membrane lipid PC during seed development and resulted in decreased residual HFA in seed PC. Consequently, seed germination potential improved in the RcPLCL1 lines compared to the RcFAH line. This result further supports a previous notion that the unusual HFA in the membrane lipid could have destructive effects on seed viability (van Erp et al., 2011; Hu et al., 2012; Snapp et al., 2014). Although RcPLCL1 was able to increase HFA accumulation in TAG, the total FA contents in RcPLCL1-expressing seeds remained largely at the same levels as in RcFAH-only seeds. Other mechanisms such as RcPDAT1 and RcDGAT2, which have been demonstrated in HFA-expressing Arabidopsis seed (Burgal et al., 2008; van Erp et al., 2011), will be required to restore the oil content to the non-transgenic levels in camelina. The decreased oil accumulation in HFA-producing seed is mainly due to feedback inhibition on fatty acid synthesis caused by inefficient incorporation of HFA onto TAG (Bates et al., 2014). Multiple mechanisms are at work to remove HFA from PC (e.g., PLA2, PDCT, and PLC) and incorporate onto TAG (e.g., DGAT and PDAT) (Burgal et al., 2008; van Erp et al., 2011; Hu et al., 2012; Bayon et al., 2015), systematic approaches will be needed to increase efficiencies of HFA synthesis and assembly.

Hydroxy fatty acids are a valuable renewable source for many industrial products. Producing these unusual fatty acids in transgenic oilseed crops is an attractive way but has met great challenges to achieve large amounts of accumulation in engineered seeds. One of the bottlenecks in HFA metabolism is an inefficient flux from PC, the site of synthesis, to storage TAG. We show here that co-expressing the fatty acid hydroxylase with a phospholipase C-like protein from castor, a native HFA accumulator, may facilitate the transfer of HFAs from PC to DAG, thus increase their accumulation in TAG of transgenic camelina. The reduced residual HFAs on the membrane lipids may also alleviate the potential detrimental effects of these unusual fatty acids on seed germination ability. Our results advanced the knowledge of lipid metabolism in seed and will help design efficient strategies to engineer high levels of HFAs in transgenic oilseeds such as the industrial crop *C. sativa*.

## Author Contributions

NA and CL designed the research and wrote the manuscript. NA conducted the experiments.

## Conflict of Interest Statement

The authors declare that the research was conducted in the absence of any commercial or financial relationships that could be construed as a potential conflict of interest.

## Footnotes

1 https://plantfadb.org/

2 https://www.ncbi.nlm.nih.gov/

3 http://www.arabidopsis.org/

4 https://prosite.expasy.org/
