# Supplementary material for: A Phospholipase C-Like Protein From Ricinus communis Increases Hydroxy Fatty Acids Accumulation in Transgenic Seeds of Camelina sativa
Source: Front Plant Sci. 2018 Nov 1;9:1576. doi: 10.3389/fpls.2018.01576 (PMC6221933; doi:10.3389/fpls.2018.01576)
Supplement: Supplementary file 1 [file Data_Sheet_1.docx]

Table S1. Expression data (FPKM) for putative Phospholipase C genes in castor. Each gene is listed along with its closest homologue in Arabidopsis according to the pairwise amino acid identity. Data are obtained from Brown et al., 2012.

| Gene  name | Gene ID | Arabidopsis homolog | Amino acid identity (%) | Endosperm  Stage II/III | Endosperm  Stage IV/V | Germinating seed | Leaf | Male flowers |
| --- | --- | --- | --- | --- | --- | --- | --- | --- |
| RcPLC-Like 1 | 30115.m001244 | At5g67130 | 69 | 49.50 | 38.33 | 43.21 | 31.74 | 24.53 |
| RcPLC-Like 2 | 29801.m003166 | At1g49740 | 71 | 9.56 | 24.59 | 5.31 | 6.26 | 23.92 |
| RcPLC-Like 3 | 29847.m000250 | At1g13680 | 63 | 2.55 | 1.34 | 1.00 | 74.04 | - |
| RcPLC-Like 4 | 30174.m009086 | At1g13680 | 64 | 1.10 | 1.63 |  | - | 244.03 |
| RcPLC-Like 5 | 29847.m000249 | At1g13680 | 62 | 0.58 |  | - | 1.50 | - |
| RcPLC-like 6 | 29756.m000515 | At4g48690 | 64 | - | - | 12.43 | 64.21 | 15.78 |
| RcNPC3 | 30148.m001447 | At3g03530 | 63 | - | - | - | - | 0.83 |
| RcNPC6 | 30147.m014488 | At3g48610 | 83 | 14.63 | 8.31 | 8.31 | 45.04 | 65.71 |


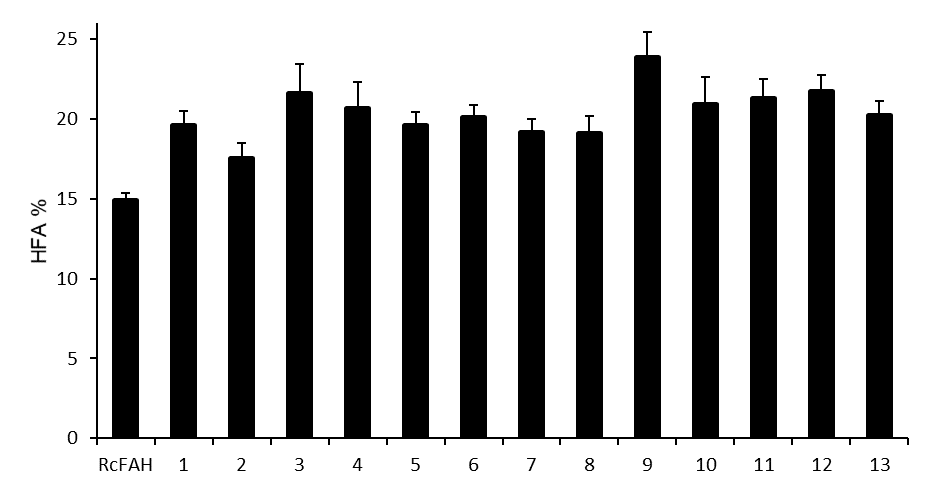


*

*

*

*

*

*

*

*

*

*

*

*

*

Fig. S1. Hydroxy fatty acid content of RcPLCL1 T1 lines. Data represent the average ± SD of highest HFA levels of single seeds from each line. Two-tailed Student’s *t* test. *, P<0.01.

Fig. S2. Fatty acid profiles of AtPLCL1 transgenic lines compared with RcFAH. Data for AtPLCL1 represent average ± SD of all 14 transgenic lines containing the highest HFA levels determined by analyzing single seeds. Two-tailed Student’s *t* test was performed to analyze the significant differences.


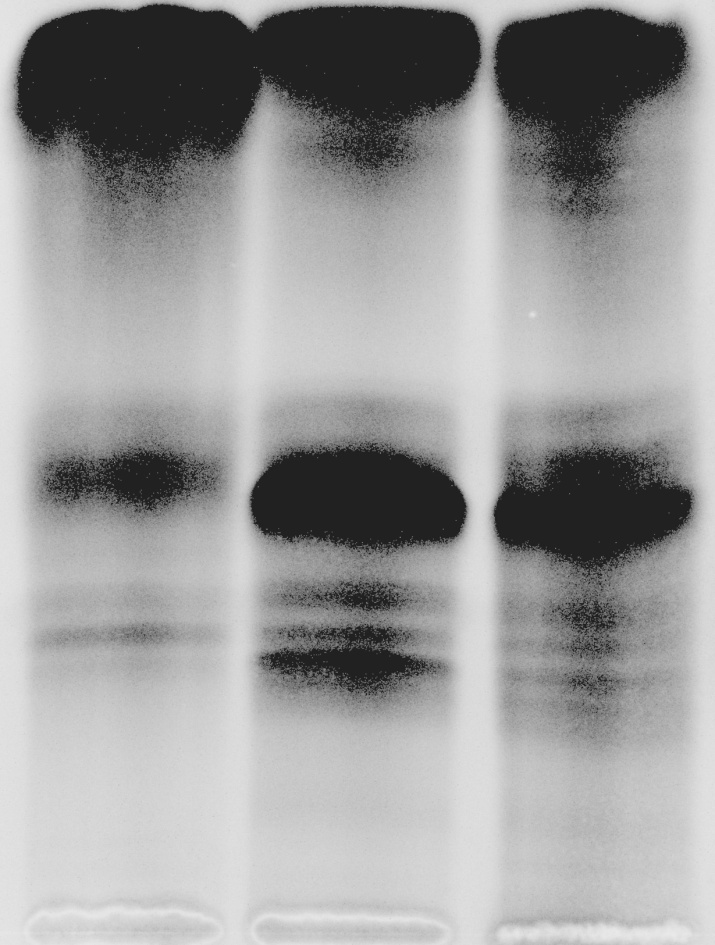


1 OH TAG

2 OH TAG

Origin

RcFAH

RcPLCL1

Suneson

TAG

Fig. S3. TLC separation of seed lipids and their molecular species in transgenic camelina lines RcPLCL1 and RcFAH. Suneson, untransformed control.
